# Effects of Hsp90 Inhibitor Ganetespib on Inhibition of Azole-Resistant *Candida albicans*

**DOI:** 10.3389/fmicb.2021.680382

**Published:** 2021-05-20

**Authors:** Rui Yuan, Jie Tu, Chunquan Sheng, Xi Chen, Na Liu

**Affiliations:** ^1^Key Laboratory of Synthetic and Natural Functional Molecule of the Ministry of Education, College of Chemistry and Materials Science, Northwest University, Xi'an, China; ^2^School of Pharmacy, Second Military Medical University, Shanghai, China

**Keywords:** Hsp90, *Candida albicans*, antifungal activity, ganetespib, drug resistance

## Abstract

*Candida albicans* is the most common fungal pathogen. Recently, drug resistance of *C. albicans* is increasingly severe. Hsp90 is a promising antifungal target to overcome this problem. To evaluate the effects of Hsp90 inhibitor ganetespib on the inhibition of azole-resistant *C. albicans*, the microdilution checkerboard method was used to measure the *in vitro* synergistic efficacy of ganetespib. The XTT/menadione reduction assay, microscopic observation, and Rh6G efflux assay were established to investigate the effects of ganetespib on azole-resistant *C. albicans* biofilm formation, filamentation, and efflux pump. Real-time RT-PCR analysis was employed to clarify the mechanism of antagonizing drug resistance. The *in vivo* antifungal efficacy of ganetespib was determined by the infectious model of azole-resistant *C. albicans*. Ganetespib showed an excellent synergistic antifungal activity *in vitro* and significantly inhibited the fungal biofilm formation, whereas it had no inhibitory effect on fungal hypha formation. Expression of azole-targeting enzyme gene *ERG11* and efflux pump genes *CDR1, CDR2*, and *MDR1* was significantly down-regulated when ganetespib was used in combination with FLC. In a mouse model infected with FLC-resistant *C. albicans*, the combination of ganetespib and FLC effectively reversed the FLC resistance and significantly decreased the kidney fungal load of mouse.

## Introduction

Invasive fungal infections (IFIs) are emerging as a severe threat in the clinic due to the increasing number of immune-impaired patients (Miceli et al., 2011; Brown et al., 2012). It is estimated that fungi killed 1.5 million individuals per year (Denning and Bromley, 2015; Enoch et al., 2017; Bassetti et al., 2018). *Candida albicans* (*C. albicans*) is the most common fungal pathogen in IFIs. Currently, only three classes of antifungal drugs are available for the treatment of infectious *C. albicans*: polyenes (e.g., amphotericin B), azoles (e.g., fluconazole), and echinocandins (e.g., caspofungin). Azoles are the first-line clinical drugs used to treat IFIs. Extensive and prophylactic use of antifungal agents, especially azoles, could easily lead to the emergence of fungal resistance, which has become a serious concern (Odds, 2005; Perfect, 2017). The resistance problem is particularly severe in *Candida* species (Revie et al., 2018). Thus, there is an urgent need to explore new treatments for resistant candidiasis.

Molecular mechanisms of drug resistance to azoles mainly include drug target (Erg11) alteration, Erg11overexpression, and efflux pump overexpression (Cuenca-Estrella, 2014; Wu et al., 2017; Lee et al., 2020). In addition, the modulation of stress responses is inextricably linked to fungal resistance. Heat shock protein 90 (Hsp90), an essential molecular chaperone in all eukaryotes, regulates the form and function of diversified client proteins (Li and Buchner, 2013; Taddei et al., 2014). Hsp90 in fungi controls stress response and enables drug resistance (Cowen, 2013; Tiwari et al., 2015). Hsp90 also acts as the enigmatic thermal sensor to govern morphological transformation in *C. albicans*, which in turn causes biofilm formation-related resistance (Cowen and Lindquist, 2005; Nett et al., 2011; Whitesell et al., 2019). The structure of nucleotide-binding domain (NBD) of *C. albicans* Hsp90 was reported, which is similar to the structure of human Hsp90 NBD (Huang et al., 2020). Targeting Hsp90 is a promising antifungal strategy to find new antiresistant agents. However, effective antifungal Hsp90 inhibitors, especially with *in vivo* antifungal activity, are still rather limited.

In this study, we evaluated the antifungal activities of four Hsp90 inhibitors. Among them, ganetespib showed the best synergistic antifungal activity both *in vitro* and *in vivo*, and its antiresistant mechanism was preliminarily clarified. The therapeutic potential of antifungal sensitizer targeting Hsp90 against azole-resistant fungi was confirmed as a promising strategy to develop novel antifungal agents.

## Materials and Methods

### Strains, Culture, and Agents

*Candida tropicalis* (*C. tropicalis*) 5008, *C. albicans* (strain numbers: 0304103 and 7781), *Cryptococcus neoformans* (*Cry.neoformans*), and *Candida glabrata* (*C. glabrata*) 9703 were provided by Changzheng Hospital of Shanghai, China. *Candida auris* (*C. auris*) 0029 was provided by Fudan University of Shanghai, China. All the strains were cultivated in yeast extract–peptone–dextrose (YEPD) medium (1% yeast extract, 2% peptone, and 2% dextrose) at 30°C in a shaking incubator (200 rpm/min). All tested compounds were purchased commercially from Topscience (Shanghai) and dissolved in DMSO at 2 mg/mL as stock solutions.

### *In vitro* Synergistic Antifungal Activity Test

The *in vitro* synergistic efficacy was measured by the microdilution checkerboard method according to the reported protocol (Huang et al., 2018). Exponentially growing fungal cells were harvested and resuspended to 1 × 10^3^ CFU/mL with RPMI 1640 medium. The tested compounds were prepared in different concentrations and transferred to the 96-well plates along the abscissa. Then, FLC was serially double-diluted into the 96-well plates along the ordinate. The azole-resistant *C. albicans* suspension containing the drug combinations was incubated at 35°C for 48 h. Then, the OD_630_ was measured by a spectrophotometer, and the synergistic inhibition efficacy was calculated using the fractional inhibitory concentration index (FICI). Each compound was tested in triplicate. FICI < 0.5 indicates the synergistic effect, FICI > 4 indicates the antagonistic effect, and 0.5 ≤ FICI ≤ 4 indicates irrelevant.

### Cell Viability Assay

*C. albicans* cells during the exponential growth phase were harvested and resuspended in fresh YPD liquid medium to an OD_600_ of about 0.40; then, the cells were diluted five times to six different concentrations in 96-well plates. About 3 μL of *C. albicans* cell suspension at different concentrations was coated on the surface of the YPD solid medium plate containing different concentrations of compounds. Then, the YPD solid medium plate was incubated at 30°C for 24 h. The growth of *C. albicans* cell colony was photographed.

### Biofilm Formation Assay

The assay was performed according to the reported protocol (Tu et al., 2019). Exponentially growing *C. albicans* 0304103 cells were harvested and diluted in RPMI 1640 medium to a concentration of 1 × 10^6^ CFU/mL. The fungal cells were transferred to the 96-well culture plates and incubated at 37°C. After 1.5 h of adhesion, the RPMI 1640 medium was aspirated to remove the non-adherent cells. Different concentrations of FLC and ganetespib were added, and the cells were further incubated at 37°C for 24 h. Then, the XTT/menadione reduction assay was used to examine the formation of biofilms. The assay was performed in triplicate.

### Filamentation Assay

The assay was performed according to the reported protocol (Ji et al., 2020). Exponentially growing *C. albicans* 0304103 were harvested and diluted in Spider medium to a concentration of 1 × 10^6^ CFU/mL. Different concentrations of FLC and ganetespib were added to the 24-well culture plate, and the plates were incubated at 37°C for 3 h. The differences in microscopic observation studies between groups were recorded on the Axio Observer D1 inverted microscope (Carl Zeiss, Inc. Thornwood, NY).

### Rh6G Efflux Assay

Exponentially growing *C. albicans* 0304103 cells were harvested and diluted in YEPD medium (8 mL). Different concentrations of FLC and ganetespib were added and incubated at 35°C for 16 h. Then, fungal cells were harvested, diluted with phosphate-buffered saline (PBS), and incubated at 35°C for 2 h, and 10 μM of Rh6G was added. After further incubation at 35°C for 30 min, 2 mM of D-glucose was added to each group. Then, the fluorescence intensity of each group was measured at 0, 20, 40, and 60 min to investigate the amount of Rh6G efflux. The assay was performed in triplicate.

### *In vivo* Antifungal Potency

Female ICR mice (4–6 weeks old and weighing 18–22 g) from the Shanghai Experimental Animal Center were housed and fed. Cyclophosphamide [100 mg/kg, in normal saline (NS)] was intraperitoneally injected to destroy the immune system of mice. After 24 h, the mice were inoculated via the tail vein with 0.2 mL of yeast suspension of *C. albicans* 0304103 (1 × 10^6^ CFU/mL). The infectious mice were divided into four groups and treated daily with saline, FLC (0.3 mg/kg, in NS), ganetespib group (25 mg/kg, suspended in NS with 1.5% glycerin and 0.5% Tween 80), and the coadministration of FLC and ganetespib until the 5th day. On day 6, all the mice were euthanized and dissected; then their left kidneys were homogenized in NS (1 mL) and diluted in different concentrations of normal saline (NS). The dilutions of kidney homogenates were inoculated on sabourauds agar (SDA) plates containing chloromycetin (100 μg/mL). The number of CFU/mL of the kidney tissue was counted to calculate the fungal burden. The differences between the groups were analyzed by analysis of variance (ANOVA).

### Real-Time RT-PCR Analysis

The analysis was performed using the reported protocol with some modifications (Han et al., 2020). Exponentially growing *C. albicans* 0304103 cells were harvested and diluted in YEPD medium at a concentration of 1 × 10^6^ CFU/mL. Different concentrations of FLC and ganetespib were added, and the blank group was made without any compound. After incubations at 30°C for 24 h, the total RNA of fungal cells were extracted according to the manufacturer's instructions (RNeasy Plant Mini kit, QIAGEN, Germany), and cDNA of cells were obtained according to the reverse transcription kit (TaKaRa, Biotechnology, China). Real-time RT-PCR was performed on LightCycler Real-time PCR system (Roche diagnostics, GmbH Mannheim, Germany) according to the protocol of the PCR amplification kit. The fluorescence change of SYBR Green I and the circulating threshold (CT) were measured (fluorescence indicator: SYBR Green I, internal control gene: ACT1). The formula 2(-ΔΔCT) was used to calculate the changes in the gene expression level, compared with ACT1. The assay was performed in triplicate. The primers are shown in Table 1.

**Table 1:** Primers for real-time RT-PCR (5′ to 3′).

**Name**	**Sequence**
*ERG11*-F	ACTCATGGGGTTGCCAATGT
*ERG11*-R	GAGCAGCATCACGTCTCCAA
*CDR1*-F	TCCACGGTCGTGAATTCCAATGTG
*CDR1*-R	GCCAGCAACAGGACCAGCTTC
*CDR2*-F	GCTACTGCCATGTCACTCTCCAC
*CDR2*-R	GGACAACTGTGCTTCCAGGAGTAG
*MDR1*-F	CCACTGGTGGTGCAAGTGTT
*MDR1*-R	TCGTTACCGGTGATGGCTCT
*ALS1*-F	GTGTCGGTTGTCAGAAGAGC
*ALS1*-R	TTGTTCACGTTGAGCCATGG
*ALS3*-F	ACTTTGTGGTCTACAACTTGGG
*ALS3*-R	CCAGATGGGGATTGTAAAGTGG
*HWP1-F*	CTGAACCTTCCCCAGTTGCT
*HWP1-R*	CGACAGCACTAGATTCCGGA
*EAP1*-F	TCCTACACGACTGACACTGC
*EAP1*-R	TGACACCCGTAGTTACTGCTG
*BCR1-F*	TCCTTTACGTGCACCACCTC
*BCR1-R*	ATGCCGACGATTCAGCTGAT
*ACE2-F*	ACTTTGTGGTCTACAACTTGGG
*ACE2-R*	CCAGATGGGGATTGTAAAGTGG
*RLM1-F*	GTGCCTGCGAATGTTCCAAA
*RLM1-R*	TGCATTGCTTCCTCCTGTCA
*ZAP1-F*	TACCGCGACTACAAACCACC
*ZAP1-R*	TGCCCCTGTTGCTCATGTTT
*ACT1*-F	GGTTTGGAAGCTGCTGGTAT
*ACT1*-R	ACCACCAATCCAGACAGAGT

## Results

### *In vitro* Antifungal Activity of Hsp90 Inhibitor Ganetespib

Four commercial Hsp90 inhibitors AUY922, ganetespib, PU-H71, and CH5138303 were selected to test the antifungal activities. The antifungal activities of Hsp90 inhibitors used alone or in combination with FLC are listed in Table 2. When used alone, four Hsp90 inhibitors had no direct effect on the growth of azole-resistant *C. albicans* (MIC_80_ > 64 μg/mL). When used in combination with FLC, FICI of AUY922, ganetespib, PU-H71, and CH5138303 was 0.039, 0.023, 2.000, and 0.500, respectively. Among them, AUY922 and ganetespib showed excellent synergistic activities. Time–growth curve revealed that the growth of azole-resistant *C. albicans* was inhibited obviously using the combination of ganetespib and FLC (8+8 μg/mL, Supplementary Figure 1). Hence, we also tested their synergistic activities against the other four azole-resistant clinically isolated *C. tropicalis, C. albicans, C. auris*, and *C. glabrata* strains. The results showed that the FICI of ganetespib was 0.039 and 0.035 against *C. tropicalis* and *C. albicans*, respectively, and ganetespib has no synergistic effect with FLC against *C. auris* and *C. glabrata* (Table 3). Furthermore, ganetespib showed moderate antifungal activity against *C. neoformans in vitro* (MIC_50_ = 8 μg/mL, Supplementary Table 1).

**Table 2:** *In vitro* antifungal activity of Hsp90 inhibitors used alone or in combination with FLC against azole-resistant *C. albicans* strain (0304103).

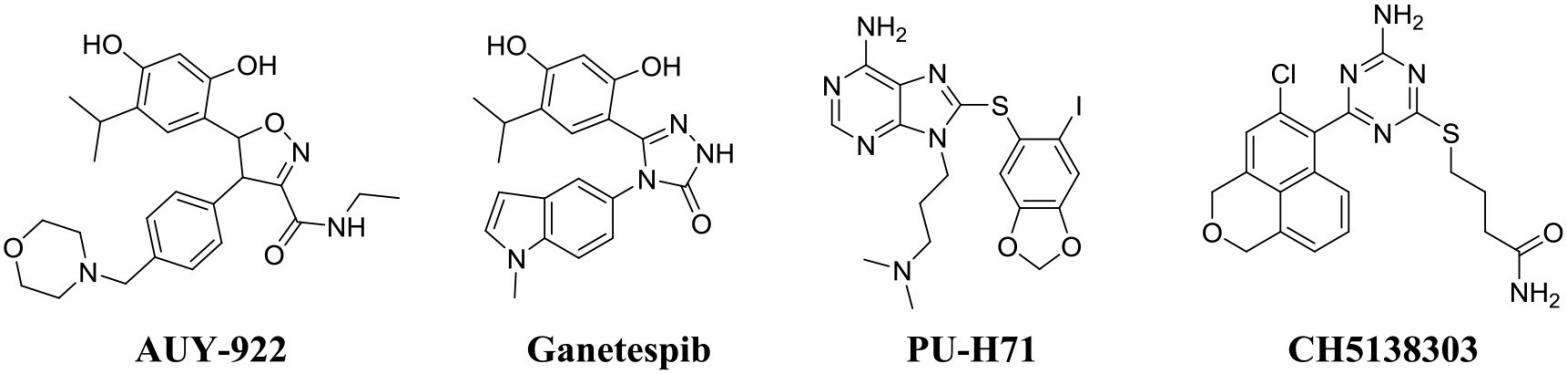				
**Compounds**	**Azole-resistant** ***C. albicans*** **0304103**			
	**MIC_80_** **(μg/mL)**	**FIC_FLC_** **(μg/mL)**	**FIC_compd._** **(μg/mL)**	**FICI^*a*^**
AUY-922	>64	2	0.5	0.039
Ganetespib	>64	1	0.5	0.023
PU-H71	>64	>64	>64	2.000
CH5138303	>64	16	16	0.500

a *Synergism was defined by FICI of ≤ 0.5 and > 4, respectively. An FICI > 0.5 but < 4 is considered irrelevant*.

**Table 3:** *In vitro* antifungal activity of ganetespib used alone or in combination with FLC against azole-resistant clinically isolated strains.

**Strains**	**MIC_80_** **(μg/mL)**	**FIC_FLC_** **(μg/mL)**	**FIC_compd_** **(μg/mL)**	**FICI**
*C. tropicalis* 5008	>64	0.5	2	0.039
*C. albicans* 7781	>64	0.25	2	0.035
*C. auris* 0029	>64	32	64	1.500
*C. glabrata* 9703	>64	16	8	1.125

### Ganetespib Affected Cell Viability

**Figure 1:**
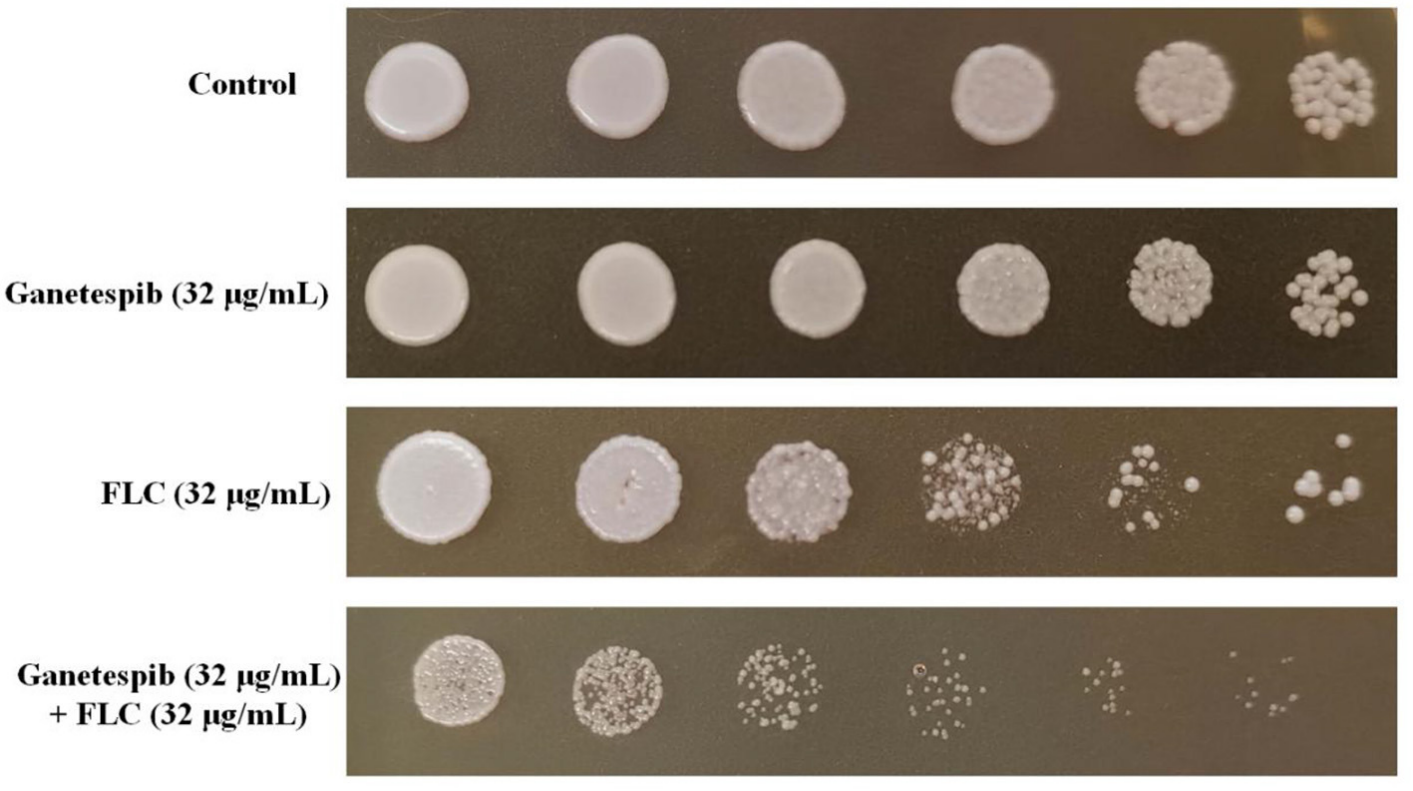
Inhibitory effect of different compounds on the cell viability of *C. albicans* 0304103. Five-fold dilutions of cells (from 1 × 10^6^/mL) were spotted on YPD.

Since the ganetespib showed an excellent synergistic antifungal activity, the inhibitory effect of cell viability was then evaluated. Cell growth was monitored in the plate assay. As shown in Figure 1, *C. albicans* cells were treated with ganetespib (32 μg/mL), FLC (32 μg/mL), as well as in combination of ganetespib and FLC (32+32 μg/mL). Compared with the blank control group, the ganetespib group (32 μg/mL) had little effect on the cell growth, and the FLC group (32 μg/mL) showed slightly reduced cell growth. In contrast, the simultaneous influence of ganetespib and FLC (32+32 μg/mL) resulted in an obvious decrease in the cell growth of *C. albicans*.

### Comparison of the Binding Mode of Ganetespib With Human and *C. albicans* Hsp90

**Figure 2:**
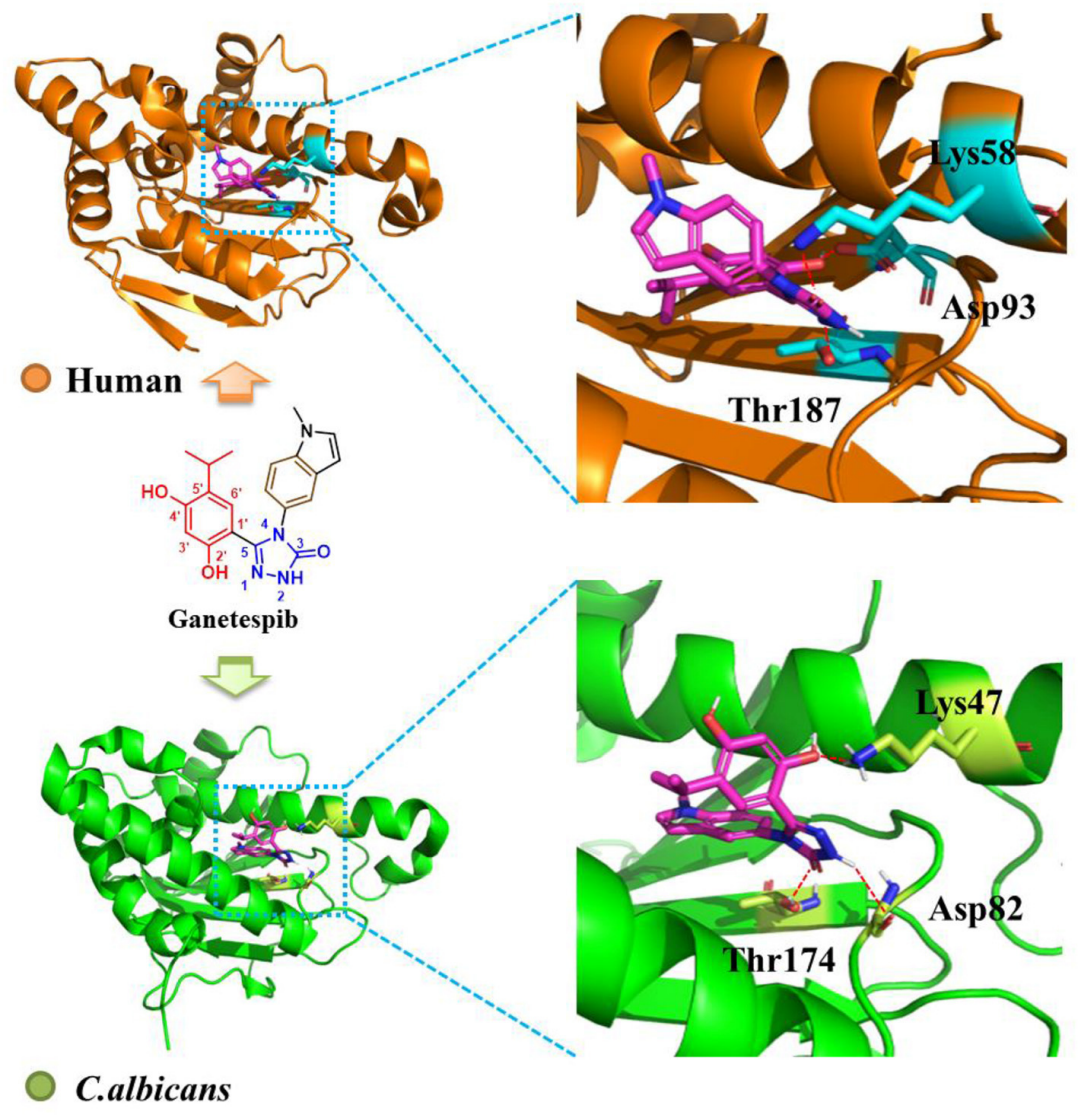
Binding mode of ganetespib with human Hsp90 (orange, PDB code: 3UTH) and *C. albicans* Hsp90 (green, PDB code: 6CJS). The carbons of ganetespib are colored in magenta, nitrogen atoms in blue, and oxygen atoms in red. Red dashed lines represent the hydrogen bonding interactions. The figure was generated using PyMol (http://www.pymol.org/).

To probe the interactions of ganetespib with *C. albicans* Hsp90, computational glide docking studies were performed by the previous docking protocols (He et al., 2018). In the crystal structure of ganetespib/human Hsp90α complex (Figure 2), the *m*-diphenol and triazolone of ganetespib were compactly bound to Hsp90α. The *m*-diphenol group interacted with Asp93 through a hydrogen bond, and the triazolone group formed two hydrogen bonds with Thr184 and Lys58. The pyrrole ring was located in the solvent-exposed region. As depicted in Figure 2, ganetespib fit well within the NBD of *C. albicans* Hsp90. The phenolic hydroxyl group of *m*-diphenol, oxygen atom, and NH of triazolone formed hydrogen bonds with Lys47, Thr174, and Asp82, respectively.

### Ganetespib Inhibited Fungal Biofilm Formation

**Figure 3:**
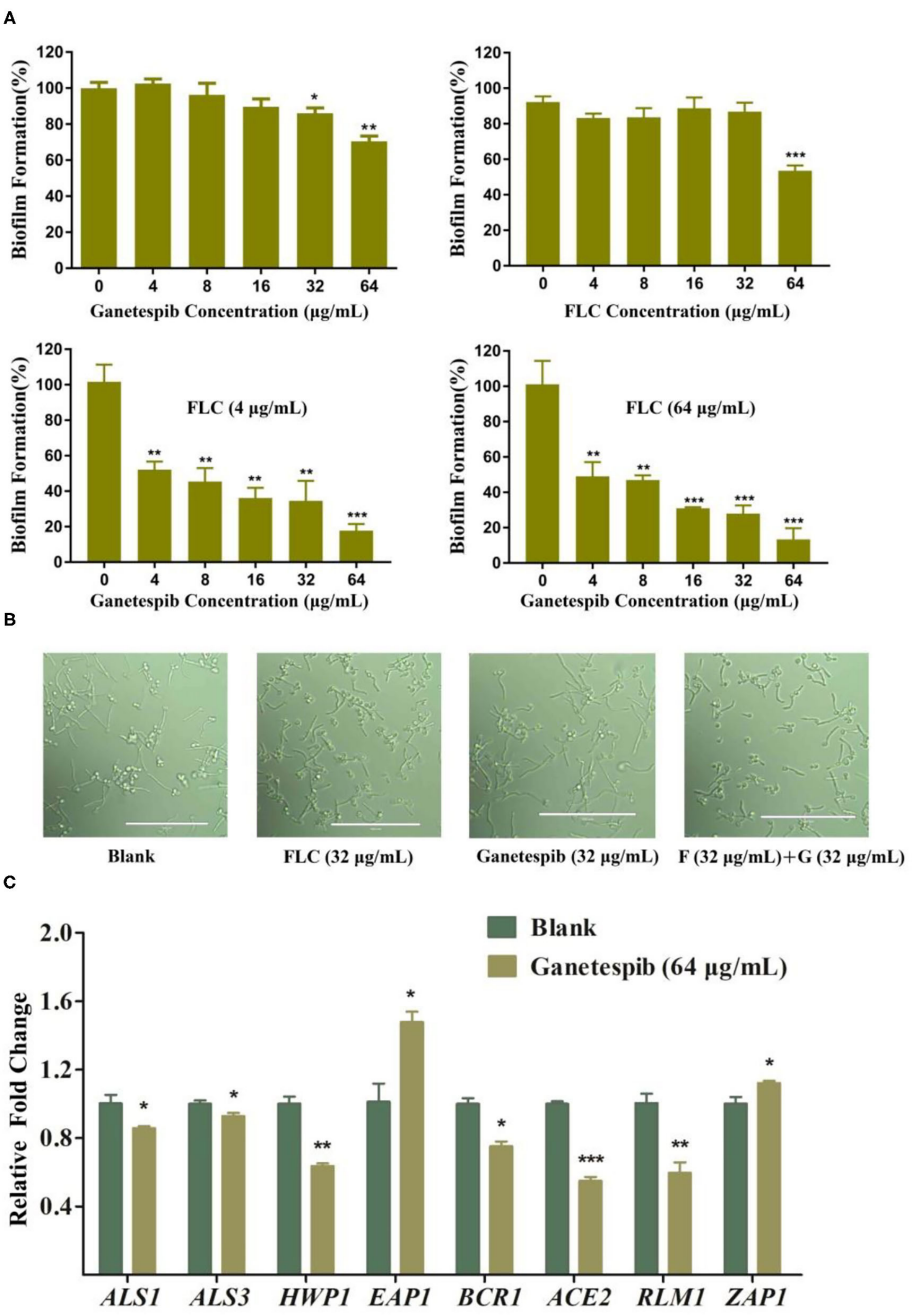
**(A)** Effect of ganetespib, FLC, or both on the *C. albicans* 0304103 biofilm formation. **(B)** Filamentation microscopic observation of *C. albicans* 0304103 treated with FLC (F), ganetespib (G), or their combination. **(C)** Expression levels of biofilm formation-related and filamentation genes (*P < 0.05, **P < 0.01, ***P < 0.001, determined by Student's *t*-test).

Formation of fungal biofilms is a critical factor in the emergence of fungal drug resistance (Kelly et al., 2004; Nobile et al., 2006a,b; Nobile et al., 2009; Liu and Filler, 2011; Roudbarmohammadi et al., 2016; Araujo et al., 2017; Oliveira-Pacheco et al., 2018). Therefore, a biofilm formation assay was performed to clarify the mechanism of antagonizing drug resistance. The result showed that ganetespib significantly inhibited the biofilm formation at high concentration (32–64 μg/mL). Furthermore, the synergistic effect of the inhibition of biofilm formation was investigated using the treatment of ganetespib in combination with FLC. The result revealed that biofilm formation was effectively inhibited at 4 μg/mL of ganetespib and FLC, respectively (*P* < 0.01), and nearly 80% inhibition could be achieved at 64 μg/mL of ganetespib in combination with 4 μg/mL of FLC (*P* < 0.001, Figure 3A). However, the growth of fungal hypha was not inhibited by the ganetespib or FLC used alone or in combination at the concentration of 32 μg/mL (Figure 3B). To further investigate the mechanism of inhibition of biofilm formation, the expression of eight biofilm-related genes was evaluated by the real-time RT-PCR. As shown in Figure 3C, *ALS1, ALS3, HWP1, BCR1, ACE2*, and *RLM1* were down-regulated, while *EAP1* and *ZAP1* were up-regulated.

### Ganetespib Obstructed Drug Resistance by Down-Regulating the Expression of Target Enzyme and Efflux Pump-Related Genes

**Figure 4:**
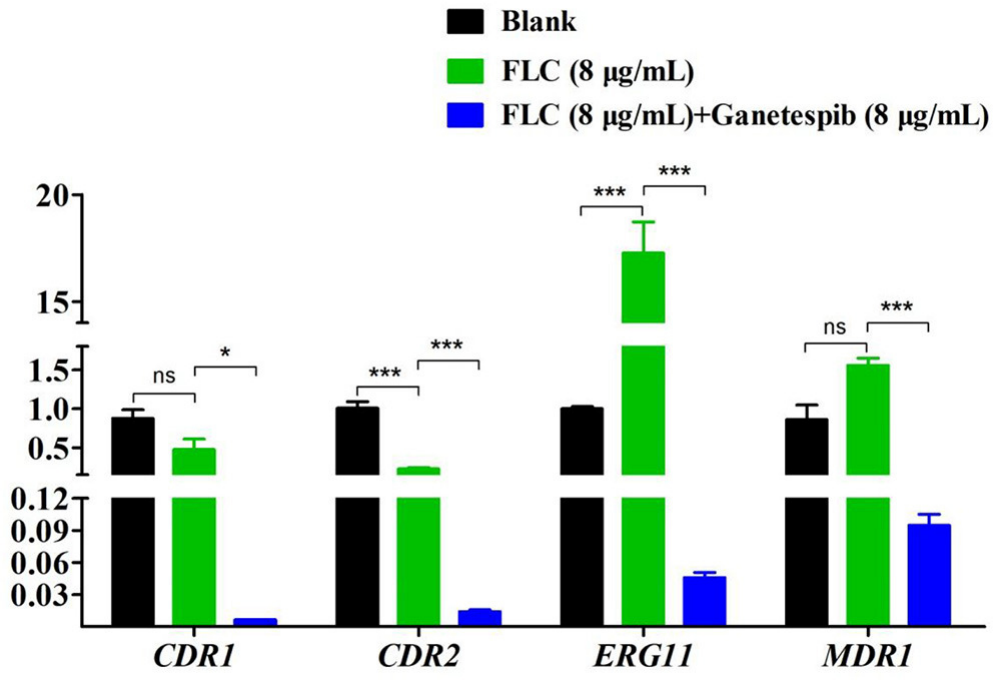
Relative expression levels of azole resistance-related genes of *C. albicans* 0304103 after the treatment of ganetespib (*P < 0.05, ***P < 0.001, determined by Student's *t*-test).

The expression of FLC target gene *ERG11* was evaluated by the real-time RT-PCR. As shown in Figure 4, under the pressure of FLC, the expression of *ERG11* was obviously up-regulated. Interestingly, under the combinational action of FLC and ganetespib, the expression of *ERG11* was dramatically down-regulated. The overexpression of the efflux pump is a key factor of azole resistance in most fungal pathogens. ATP-binding cassette (ABC) superfamily and the major facilitator (MF) superfamily are the two main classes of efflux pumps contributing to azole resistance (Lee et al., 2020). In *C. albicans*, Cdr1 and Cdr2, two homologous ABC transporters, are closely associated with azole resistance. Besides, FLC resistance is also relevant to the MF transporter Mdr1 (multidrug resistance 1). Therefore, the expression of *CDR1, CDR2*, and *MDR1* was evaluated by the real-time RT-PCR. The results showed that the expression of *CDR1, CDR2*, and *MDR1* was significantly down-regulated when the FLC was used in combination with ganetespib.

### Rhodamine 6G (Rh6G) Efflux Assay

**Figure 5:**
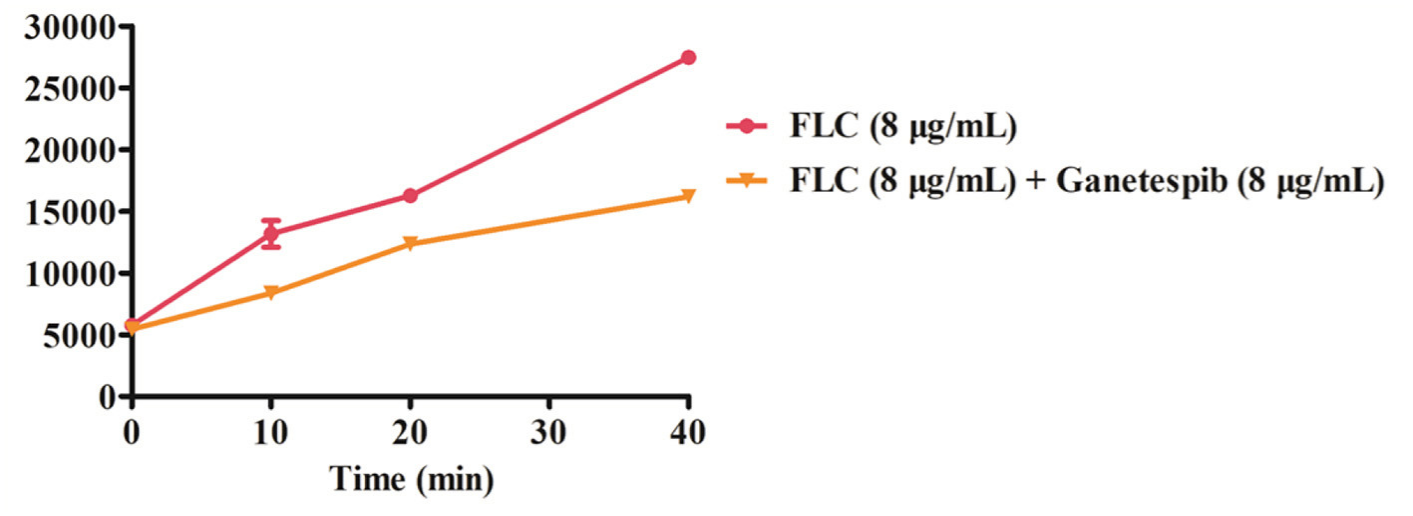
Extracellular fluorescence intensity of Rh6G treated with ganetespib.

By monitoring the fluorescence intensity, the extracellular Rh6G content was obtained. As shown in Figure 5, compared with FLC used alone, the fluorescence intensity was obviously lower when used in combination with ganetespib. The results indicated that the Rh6G excretion was decreased after the addition of ganetespib. As a result, the expression of efflux pumps was significantly declined under the action of ganetespib.

### *In vivo* Antifungal Activity of Hsp90 Inhibitor Ganetespib

**Figure 6:**
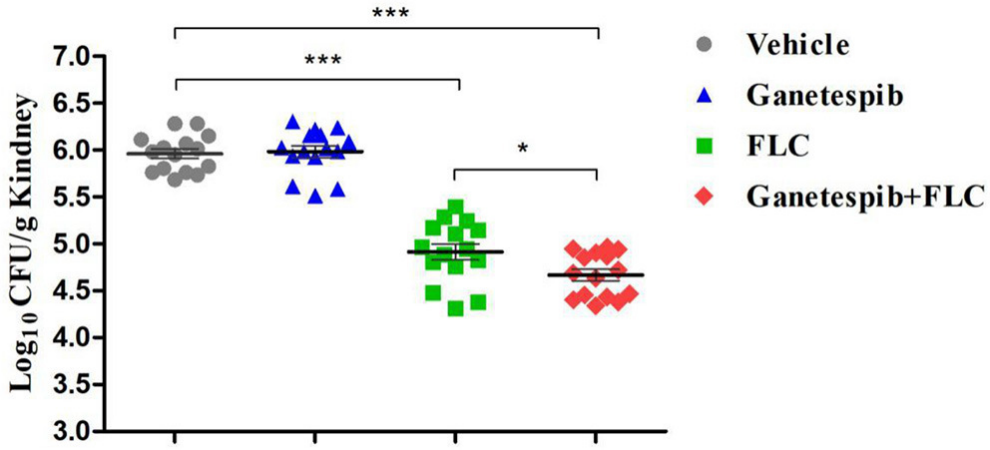
*In vivo* therapeutic efficacy of ganetespib against IFI mice model infected with azole-resistant *C. albicans* 0304103 (*P < 0.05, ***P < 0.001, determined by ANOVA).

IFI mice model was constructed to investigate the *in vivo* antifungal activity of Hsp90 inhibitor ganetespib. Count of kidney fungal load was used as the evaluation index (Figure 6). The mice were divided into four groups: vehicle group, ganetespib group, FLC group, and the combination of ganetespib and FLC group. Compared with the blank group, ganetespib (25 mg/kg) used alone had no *in vivo* activity, however, treatment in combination with ganetespib (25 mg/kg) and FLC (0.3 mg/kg) could significantly decrease the kidney fungal load of mouse (*P* < 0.001), which was better than FLC used alone (*P* < 0.05).

## Discussion

Currently, the IFIs are a serious threat to public health in the clinic. *C. albicans* is the most common pathogenic fungi of IFIs. In recent years, drug resistance of abusive use of antifungal agents is becoming a serious problem. Thus, the antifungal efficacy of current drugs for the treatment of azole-resistant *C. albicans* in the clinic is limited (Arendrup and Patterson, 2017). Therefore, the discovery of new antifungal agents against resistant fungi is highly desirable.

Hsp90, a highly conserved molecular chaperone, plays an important role in antifungal drug tolerance and resistance in *Candida spp*., and is regarded as a promising antifungal target. In this study, we assayed four Hsp90 inhibitors for the direct and synergistic antifungal activity. Ganetespib was validated to possess excellent synergistic activities when used in combination with FLC against azole-resistant *C. albicans* clinical isolates.

The crystal structures of human and *C. albicans* Hsp90 have been reported (Lee et al., 2015). We performed the docking study to compare the binding modes of ganetespib with human and *C. albicans* Hsp90. The result indicated that ganetespib fit well within the NBD of *C. albicans* Hsp90. Therefore, the cytotoxicity of ganetespib might hamper its potential application as an antifungal enhancer. Herein, *in vitro* antitumor activity and Hsp90α enzyme inhibition activity of ganetespib were also tested (Supplementary Table 2 and Supplementary Figure 2). The results indicated that ganetespib had an excellent antitumor activity against HEL cells (IC_50_ = 0.021 μM), HL60 cells (IC_50_ = 0.023 μM), and A549 cells (IC_50_ = 0.11 μM), and an excellent Hsp90α enzyme inhibition activity (IC_50_ = 0.010 μM). Thus, further structural optimization of ganetespib is required to reduce the cytotoxicity. Recently, fungal-selective HSP90 inhibitors have been reported (Huang et al., 2020; Marcyk et al., 2021). However, the improvement of selectivity seems to have little influence on reducing cytotoxicity. Thus, extensive medicinal chemistry efforts are essential to developing HSP90 inhibitors as new antifungal agents.

The mechanism of ganetespib in antagonizing drug resistance was preliminarily investigated. Ganetespib significantly inhibited the biofilm formation used alone or in combination with FLC, but the growth of fungal hypha was not inhibited. It is inferred that ganetespib inhibited the biofilm formation not through acting on hypha formation. The effects of ganetespib on the expression of biofilm-related genes indicated that the ganetespib inhibited the biofilm formation relevant to cell adhesion.

Currently, molecular mechanisms of azole resistance included drug target alteration, drug target overexpression, and efflux pump overexpression (Vila et al., 2017). Overexpression of drug target (Erg11) of azoles is common in azole-resistant *C. albicans* and leads to low drug sensitivity. Under the combinational treatment of FLC and ganetespib, the expression of *ERG11* and efflux pump genes *CDR1, CDR2*, and *MDR1* was significantly down-regulated. Though, the detailed mechanism of the drug combination in reversing *C. albicans* resistance remains to be clarified.

Taken together, this study investigated the *in vitro* and *in vivo* synergistic antifungal activities of ganetespib. Ganetespib could be used as a lead compound to develop a novel antifungal enhancer to treat resistant *Candida* infections.

## Data Availability Statement

The raw data supporting the conclusions of this article will be made available by the authors, without undue reservation, to any qualified researcher.

## Ethics Statement

The animal study was reviewed and approved by Committee on Ethics of Medicine, Navy Medical University, PLA.

## Author Contributions

NL designed the experiments. RY and JT performed the experiments and interpreted the data. NL, XC, and CS wrote the manuscript. All authors contributed to the article and approved the submitted version.

## Conflict of Interest

The authors declare that the research was conducted in the absence of any commercial or financial relationships that could be construed as a potential conflict of interest.
