# Corrigendum: Effects of Hsp90 Inhibitor Ganetespib on Inhibition of Azole-Resistant *Candida albicans*

**DOI:** 10.3389/fmicb.2021.773140

**Published:** 2021-10-19

**Authors:** Rui Yuan, Jie Tu, Chunquan Sheng, Xi Chen, Na Liu

**Affiliations:** ^1^Key Laboratory of Synthetic and Natural Functional Molecule of the Ministry of Education, College of Chemistry and Materials Science, Northwest University, Xi'an, China; ^2^School of Pharmacy, Second Military Medical University, Shanghai, China

**Keywords:** Hsp90, *Candida albicans*, antifungal activity, ganetespib, drug resistance

In the original article, there was a mistake in Figure 3 as published. In Figure 3B, the wrong image was used for the filamentation microscopic figure of *C. albicans* 0304103 treated with Ganetespib (32 μg/mL). The corrected Figure 3 appears below.

**Figure 3:**
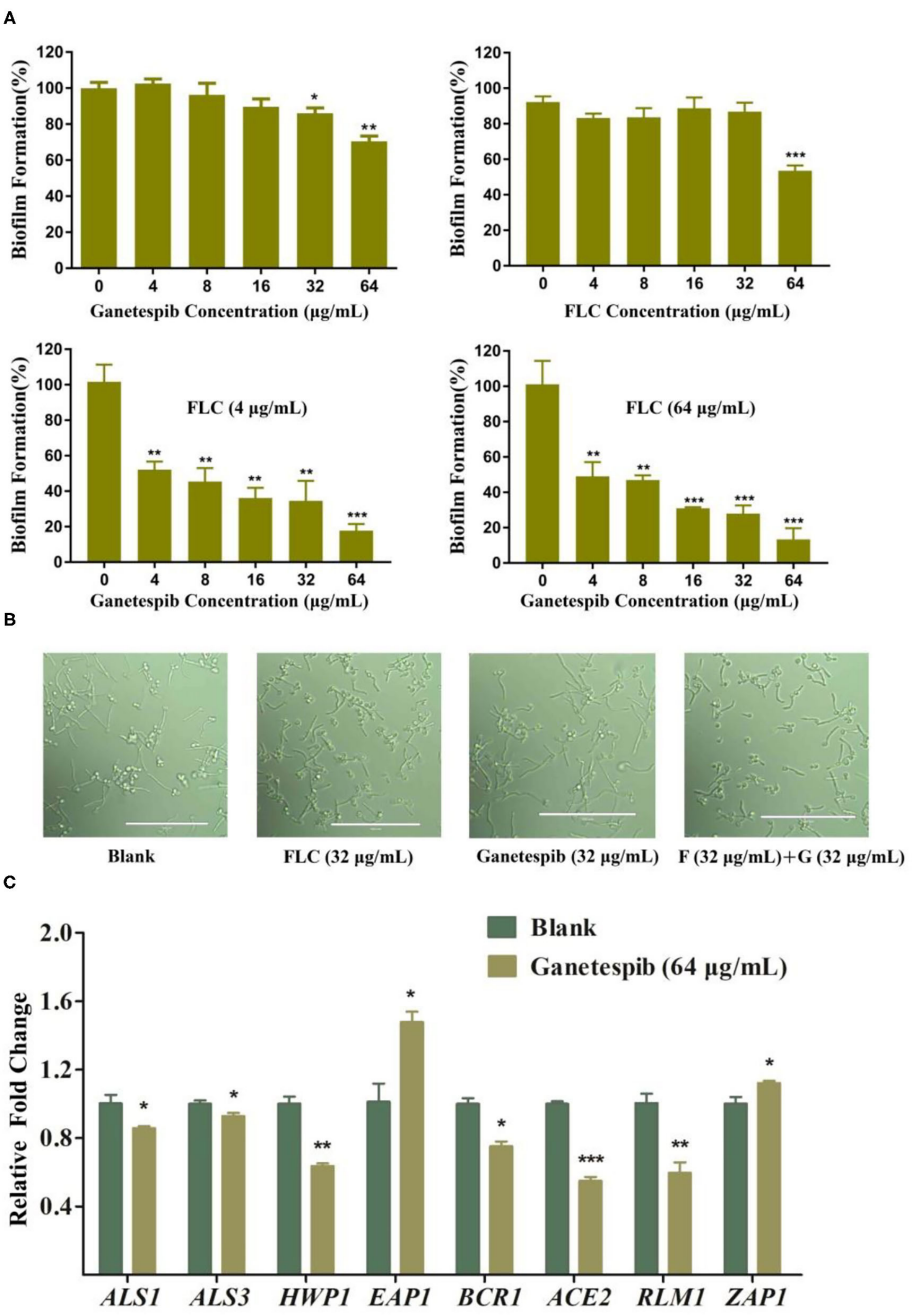
**(A)** Effect of ganetespib, FLC, or both on the *C. albicans* 0304103 biofilm formation. **(B)** Filamentation microscopic observation of *C. albicans* 0304103 treated with FLC (F), ganetespib (G), or their combination. **(C)** Expression levels of biofilm formation-related and filamentation genes (*P < 0.05, **P < 0.01, ***P < 0.001, determined by Student's *t*-test).

The authors apologize for this error and state that this does not change the scientific conclusions of the article in any way. The original article has been updated.

## Publisher's Note

All claims expressed in this article are solely those of the authors and do not necessarily represent those of their affiliated organizations, or those of the publisher, the editors and the reviewers. Any product that may be evaluated in this article, or claim that may be made by its manufacturer, is not guaranteed or endorsed by the publisher.

