# Supplementary material for: Effects of Hsp90 Inhibitor Ganetespib on Inhibition of Azole-Resistant Candida albicans
Source: Front Microbiol. 2021 May 20;12:680382. doi: 10.3389/fmicb.2021.680382 (PMC8174564; doi:10.3389/fmicb.2021.680382)
Supplement: Supplementary file 1 [file Data_Sheet_1.docx]

Supplementary Material

**Table S1 ︱** *In vitro* antifungal activity of Hsp90 inhibitor ganetespib against *Cryptococcus neoformans* (MIC_50_, μg/mL).

| **Strains** | | **Ganetespib** | | **FLC** |
| --- | --- | --- | --- | --- |
| H99 | 8 | | 0.5 | |

**Table S2 ︱** *In vitro* antitumor activity of Hsp90 inhibitor ganetespib.

| **Cancer cell lines** | **IC_50_ (μM)** |
| --- | --- |
| HEL | 0.021 ± 0.002 |
| HL60 | 0.023 ± 0.0023 |
| A549 | 0.11 ± 0.019 |


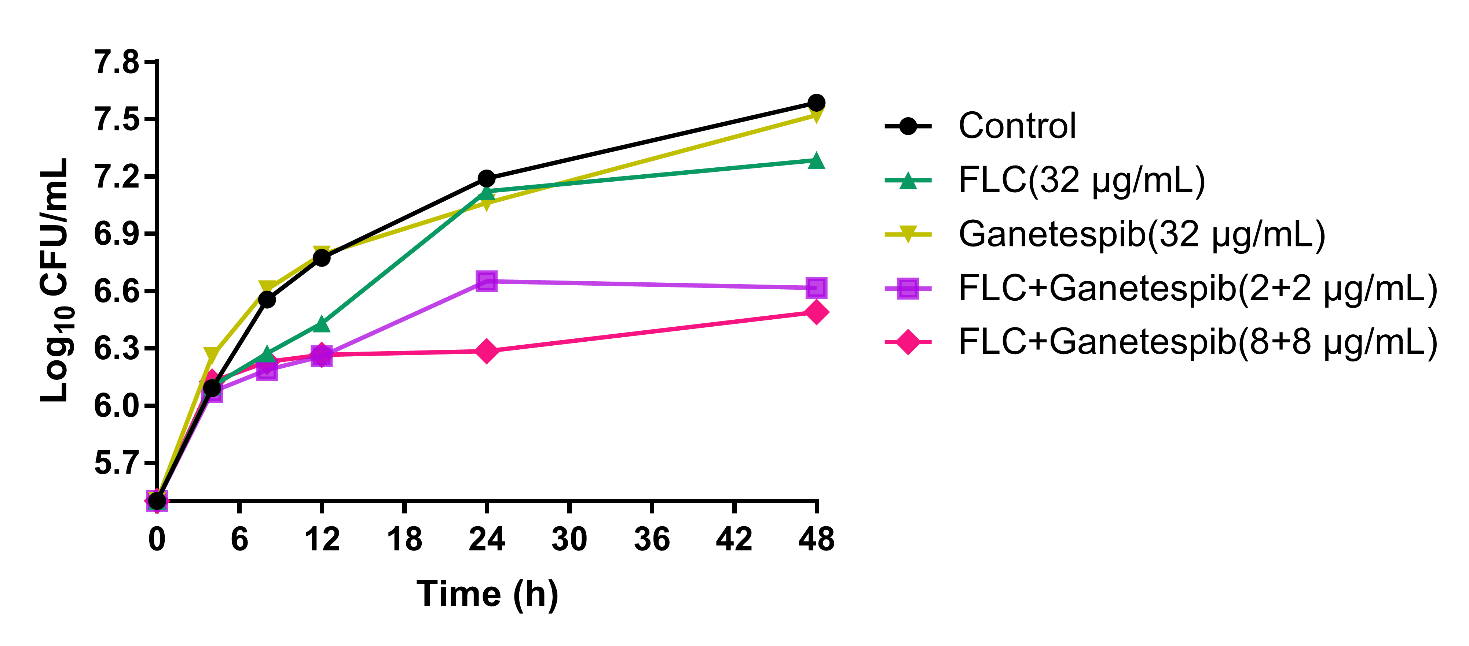


**FIGURE S1 ︱** Time-growth curve of *C. albicans* 0304103 treated with increasing concentrations of FLC, ganetespib, or their combination.


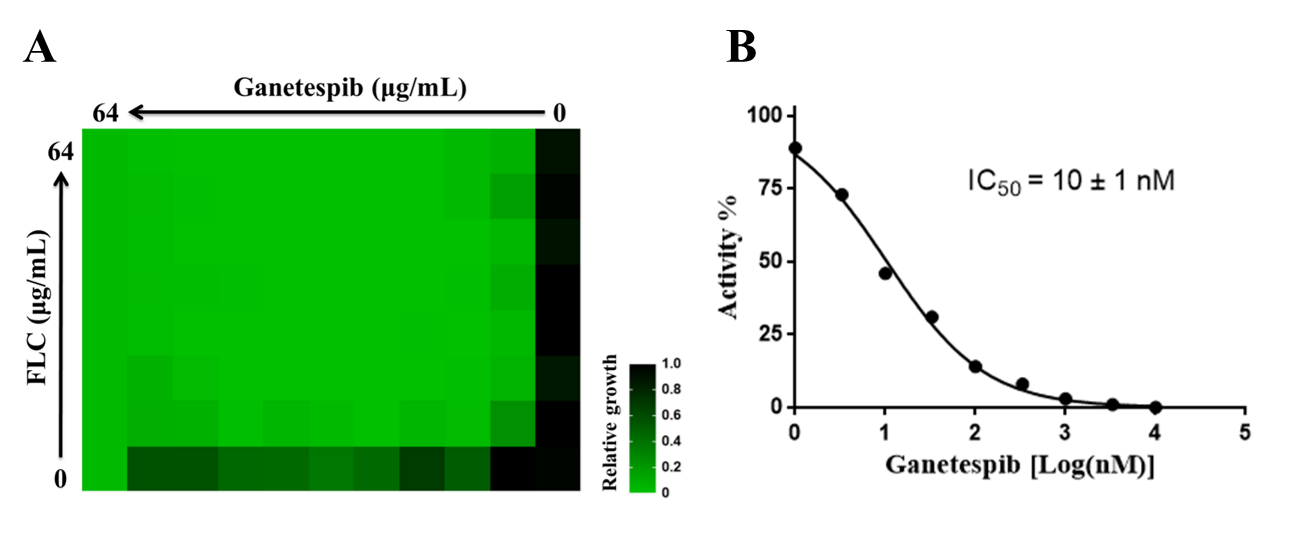


**FIGURE S2 ︱** Biological activity of ganetespib. **(A)** The synergistic activity of ganetespib in combination with FLC was displayed as a Heatmap. **(B)** Hsp90α enzyme inhibition activity of ganetespib.

Hsp90α Enzyme Test

A series of dilutions of the test compounds were prepared with 10% DMSO in assay buffer and 10 μL of the dilution was added to a 100 μL reaction so that the final concentration of DMSO is 1% in all reactions. The reactions were conducted at room temperature for 3 h in a 100 μL mixture containing assay buffer, 5 nM FITC Labeled Geldanamycin and Ganetespib. Fluorescence intensity was measured at an excitation of 485 nm and an emission of 530 nm using a Tecan Infinite M1000 microplate reader. Activity assays were performed in duplicate at each concentration. Fluorescence intensity is converted to fluorescence polarization using the Tecan Magellan6 software. The IC_50_ value was determined by using the computer software, Graphpad Prism.
